# Targeted Enrichment Sequencing for Wastewater Resistome Surveillance: A Proof-of-Concept Study Across Treatment Stages and Seasons

**DOI:** 10.3390/toxics14080650

**Published:** 2026-07-24

**Authors:** Giusy Bonanno Ferraro, David Brandtner, Carolina Veneri, Pamela Mancini, Agata Franco, Daniele Congiu, Marcello Iaconelli, Monica Monaco, Maria Del Grosso, Giulia Errico, Elisabetta Suffredini, Giuseppina La Rosa

**Affiliations:** 1 National Center for Water Safety (CeNSia), Istituto Superiore di Sanità, Viale Regina Elena 299, 00161 Rome, Italy; david.brandtner@iss.it (D.B.); carolina.veneri@iss.it (C.V.); pamela.mancini@iss.it (P.M.); agata.franco@iss.it (A.F.); daniele.congiu@iss.it (D.C.); marcello.iaconelli@iss.it (M.I.); giuseppina.larosa@iss.it (G.L.R.); 2 Department of Infectious Diseases, Istituto Superiore di Sanità, 00161 Rome, Italy; monica.monaco@iss.it (M.M.); maria.delgrosso@iss.it (M.D.G.); giulia.errico@iss.it (G.E.); 3 Department of Food Safety, Nutrition and Veterinary Public Health, Istituto Superiore di Sanità, 00161 Rome, Italy; elisabetta.suffredini@iss.it

**Keywords:** wastewater treatment plant (WWTP), resistome, probe enrichment, antimicrobial resistance genes (ARGs), antimicrobial agent categories (AACs)

## Abstract

The detection of antibiotic resistance genes (ARGs) in wastewater is commonly based on targeted PCR assays, limiting analysis to predefined genes. Consequently, the structure of wastewater resistomes often remains poorly characterised. Targeted enrichment sequencing offers an alternative approach for comprehensive resistome profiling. A panel targeting 2786 ARGs was applied to eight influent–effluent wastewater samples collected over one year from a large wastewater treatment plant in central Italy. Sequencing data were analysed at both the ARG and Antimicrobial Agent Categories (AACs) levels, and seasonal variation was explored using a ratio-based clustering approach. Overall, 743 ARGs were detected in both influents and effluents, with 84% of them. The resistome showed a strongly uneven structure, with a limited number of highly abundant ARGs accounting for ~60% of total abundance, while more than 500 genes were detected at ultra-low abundance. Thirty-five AACs were represented. Seasonal resistome profiles were generally stable, although winter samples showed a distinct enrichment pattern involving multiple ARGs, particularly tetracycline-associated ARGs. This structure was evident in influents becoming less defined in effluents. Targeted enrichment sequencing provided a broader view of the wastewater resistome, revealing a large pool of ARGs and seasonal patterns that would likely remain undetected using conventional PCR-based approaches.

## 1. Introduction

Antibiotic-resistant bacteria (ARB) and antibiotic resistance genes (ARGs) are commonly found in various environmental reservoirs, including wastewater, which represents an important pathway for the dissemination of antimicrobial resistance (AMR) into natural ecosystems [1,2,3]. Wastewater treatment plants (WWTPs) receive complex microbial communities originating from human, hospital and industrial sources, and are increasingly recognised as important interfaces for the circulation and environmental release of resistance determinants.

The growing recognition of AMR as a One Health challenge has led international organisations and regulatory frameworks to emphasise the need for environmental surveillance strategies integrating wastewater monitoring. The World Health Organisation (WHO) and the European Union (EU) have progressively highlighted the importance of extending AMR surveillance beyond clinical settings to include environmental compartments such as wastewater [4,5]. This approach has been further reinforced by the recast Urban Wastewater Treatment Directive (Directive EU 2024/3019) [6], which introduces, for the first time, a requirement for Member States to develop monitoring frameworks for AMR-related indicators in urban wastewater. Several studies have shown that ARGs are commonly found not only in wastewater influent but also in treated effluent, indicating that resistance determinants can persist throughout the treatment process [7,8,9,10]. Indeed, although WWTPs are designed to remove chemical and microbiological contaminants, they are often ineffective at completely removing ARGs [11].

Traditional approaches for detecting ARGs in wastewater mainly rely on targeted real-time PCR or digital PCR assays. Although highly sensitive and quantitative, these methods are inherently limited to a restricted number of pre-selected genes and therefore cannot capture the broader complexity of resistomes in environmental matrices. As a result, rare, emerging or low-abundant ARGs often remain undetected. In particular, low-abundance ARGs, although individually contributing little to total abundance, collectively represent a large and functionally diverse fraction of the resistome and often remain undetected. This limitation constrains the ability of current surveillance strategies to identify emerging resistance patterns and fully characterise resistome structure.

Recent advances in sequencing strategies have enabled broader resistome analyses in environmental matrices. While shotgun metagenomics provides an untargeted overview of microbial communities and resistance determinants, its sensitivity for low-abundance ARGs in complex matrices such as wastewater can be limited by sequencing depth, making targeted enrichment approaches an attractive alternative for surveillance applications.

Hybrid-capture targeted enrichment occupies an intermediate position between these two strategies. Unlike PCR or qPCR/dPCR assays, it is not restricted to a handful of pre-selected targets, and unlike shotgun metagenomics, it does not rely on sufficient sequencing depth to detect rare determinants against a complex background of non-target DNA; instead, probe-based capture of thousands of resistance markers concentrates sequencing effort on ARGs specifically, substantially increasing sensitivity for low-abundance genes without the target restriction inherent to PCR-based panels. These panels were originally developed for clinical diagnostic use, where the priority is the sensitive detection of a curated set of clinically relevant resistance determinants in relatively simple matrices. Their transfer to wastewater is not straightforward, since environmental samples are compositionally far more complex, and it remains unclear whether panels designed for clinical targets adequately capture the broader, environmentally relevant resistome, and how such data behave across treatment stages and seasonal variation. To date, hybrid-capture enrichment has been applied to wastewater matrices in only a small number of studies, focused on hospital wastewater or WWTP effluents [12,13]. To our knowledge, no previous study has evaluated this approach across both influent and effluent stages of a full treatment plant over multiple seasons. Building on this gap, the present study evaluates the feasibility of this approach for wastewater resistome profiling through a longitudinal design spanning influent and effluent samples across four seasons. This is addressed through the following specific objectives:

(i) characterise ARG diversity and abundance patterns in influent and effluent samples;
(ii) evaluate the distribution of ARGs across resistance gene categories;
(iii) explore seasonal variations in resistome composition using abundance-based and clustering-based analyses.

## 2. Materials and Methods

### 2.1. Sample Collection, Filtration and DNA Extraction

Composite 24 h influent and effluent wastewater samples were collected one per season over one year (2023) from a large municipal WWTP in central Italy, resulting in a total of eight samples. Samples were collected upstream of any treatment process for influent and downstream of the final treatment step for effluent, using an automatic refrigerated sampler. Different filtration volumes were used for influent and effluent samples to account for differences in biomass concentration and to ensure sufficient DNA recovery from treated wastewater (50 mL for influent and 500 mL for effluent samples). A slightly larger volume than the target filtration volume was collected for each sample to compensate for potential losses during transport and processing. Samples were transported to the laboratory under refrigerated conditions (+4 °C) and processed immediately upon arrival. Samples were filtered using 0.22 μm mixed cellulose ester filters (Whatman, Little Chalfont, UK), and one filtration blank was included for each sampling campaign and processed in parallel. Total DNA was extracted using the DNeasy PowerWater Kit (QIAGEN, Hilden, Germany) according to the manufacturer’s instructions, using the entire membrane filter as starting material for lysis. DNA was eluted in a final volume of 100 μL, which was then aliquoted and stored at −80 °C in single-use aliquots to avoid repeated freeze–thaw cycles for subsequent analysis. DNA quality and quantity were assessed using both the Qubit dsDNA HS fluorometric assay kit (Invitrogen, Carlsbad, CA, USA) and the Agilent^®^ High Sensitivity DNA kit (Agilent Technologies, Santa Clara, CA, USA).

### 2.2. Hybrid-Capture Enrichment and Illumina Sequencing

Targeted enrichment-based sequencing was carried out using the QIAseq xHYB AMR Panel Kit (Qiagen), which targets 2786 ARGs. The full list of targeted genes and associated resistance classes is provided in Supplementary Tables S1 and S2. Libraries were prepared using the QIAseq FX DNA library kit (Qiagen, Hilden, Germany) with unique dual index (UDI) adapters, according to the manufacturer’s instructions. The workflow included single-tube DNA fragmentation, end-repair, A-tailing, adapter ligation, bead-based purification, and library amplification using the QIAseq HiFi PCR Master Mix (Qiagen, Germantown, MD, USA). Individual libraries were then pooled and hybridised overnight with the QIAseq xHYB probe set (Qiagen, Germantown, MD, USA). Enriched fragments were captured using streptavidin-coated magnetic beads, followed by post-capture amplification. Final enriched libraries were quantified and sequenced on an Illumina NextSeq 2000 platform (Illumina, San Diego, CA, USA) using a P1 (300-cycle) kit and 2 × 150 bp paired-end chemistry. A schematic overview of the entire hybrid-capture and sequencing workflow is illustrated in Figure S1 (Supplementary Materials).

### 2.3. Bioinformatic and Downstream Data-Analysis Pipeline

Raw sequencing reads were analysed using the Microbial Genomics Module (CLC-MGM) implemented in CLC Genomics Workbench v21.0.5 (Qiagen Bioinformatics, Germantown, MD, USA). After import, reads underwent quality processing. At first, quality trimming was applied, retaining the reads with a minimum global running score (Mott algorithm) of Q15 or, after trimming, the part of the read above the threshold. Illumina adapters at read ends were sliced off, and final-length filtering was applied, discarding processed reads under 50 bp. ARG detection was performed using the Find Resistance with ShortBRED (FRSB) tool, which identifies ARG-associated peptide-markers through translational alignment of sequencing reads against a curated resistance database and normalises their abundance per sample with a modified RPKM formula that takes into account the target peptide marker length instead of the full gene length. The reference database used was QMI-AR (QIAGEN Microbial Insight—Antimicrobial Resistance database), which integrates peptide markers derived from multiple AMR databases, including CARD, ARG-ANNOT, the NCBI Bacterial Antimicrobial Resistance Reference Gene Database, and ResFinder. The FRSB output consisted of a single wide-format table containing all analysed samples. Each row corresponded to one detected ARG-associated peptide marker, together with quantitative information including raw read counts and Reads Per Kilobase per Million mapped reads (RPKM)-normalised values. Additional annotation fields described the ARG associated with each peptide marker, the corresponding antibiotic class(es), and the related resistance mechanism. ARGs associated with resistance to multiple antibiotic classes were reported with multiple annotations. All downstream analyses were performed at the ARG level. Although the ShortBRED/FRSB workflow initially reports resistance at the peptide-marker level, peptide markers were used only as intermediate technical units and were systematically aggregated to their corresponding ARGs prior to all comparative, abundance and seasonal analyses. For each sample, peptide markers associated with the same ARG were aggregated by summing their quantitative values, resulting in a single abundance value per ARG. To facilitate downstream analyses, the dataset was restructured so that each row represented the detection of a single ARG in a single sample, along with associated metadata, including raw read counts, RPKM-normalised abundance, sampling stage (influent/effluent), and season. In addition, RPKM-normalised abundances were further converted into Transcripts Per Million (TPM) values, which were used as the normalised abundance metric for all subsequent comparative analyses. To facilitate ecological interpretation, ARGs were grouped into descriptive abundance categories based on relative abundance: major ARGs (>1%), minor ARGs (0.1–1%) and ultra-minor ARGs (<0.1%). In addition, ARGs were grouped into broader Antimicrobial Agent Categories (AACs), which included conventional antibiotic classes, as well as additional antimicrobial agents, such as disinfectants/antiseptics and multiclass resistance determinants. AAC assignment was based on a manually curated classification derived from pharmacological and clinical nomenclature. ARGs associated with resistance to multiple antibiotic classes were assigned to multiple AACs accordingly. ARG abundances (TPM values) were subsequently aggregated at the AAC level to enable broader comparisons of resistome patterns across wastewater stages and seasons. During this process, manual curation was performed to verify ARGs associated with multiple resistance categories and to resolve ambiguous resistance annotations when necessary. According to the Comprehensive Antibiotic Resistance Database (CARD) ontology [14], specific classes are listed when a gene’s resistance profile is precisely documented (e.g., macrolides and lincosamides). In contrast, the multiclass designation is used for single genetic features, such as broad-spectrum efflux pumps or global regulators, that provide generalised protection across a vast range of antibiotic families.

### 2.4. Seasonality Analysis and Heatmap Construction

**Figure 1:**
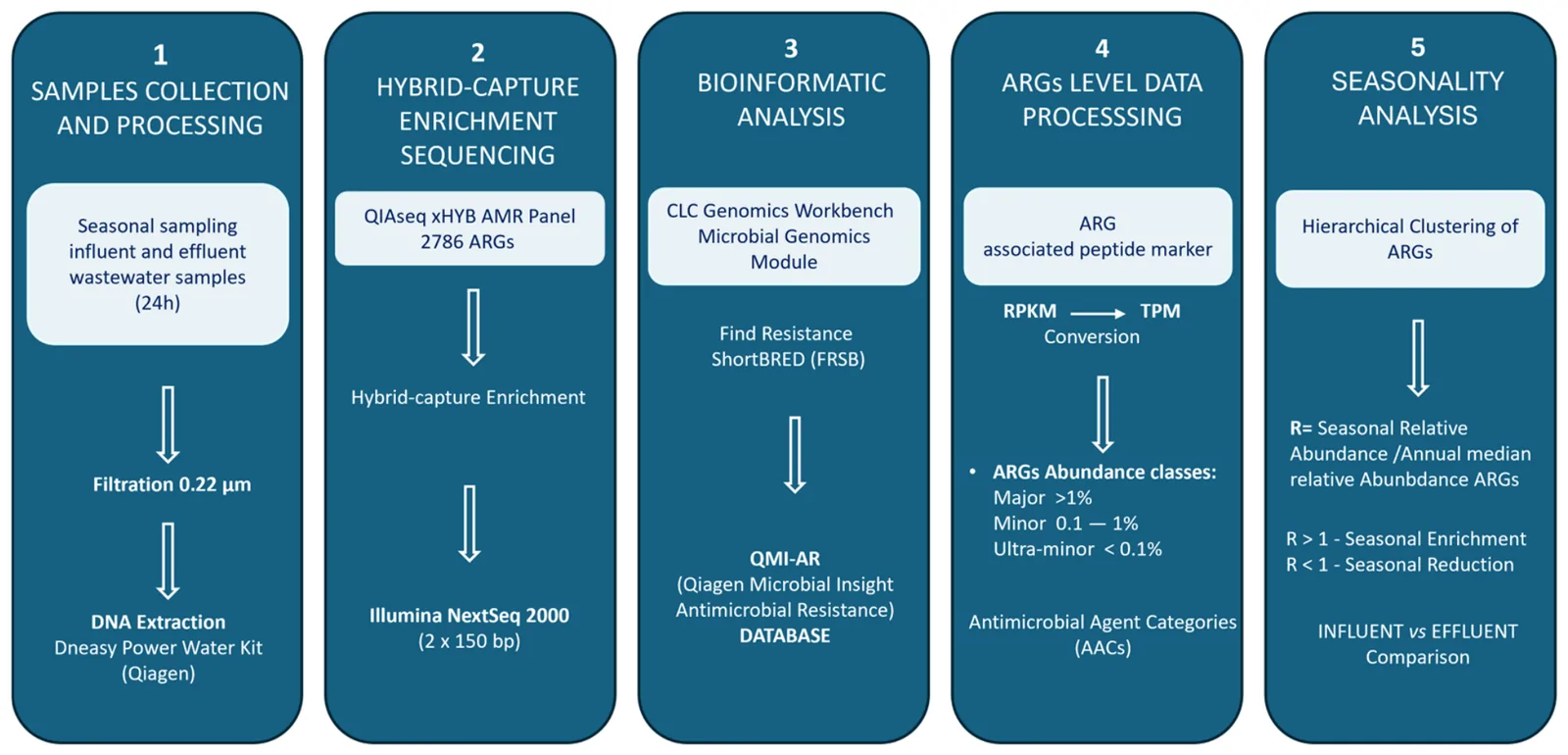
Overall experimental and analytical workflow used for wastewater resistome profiling, including: 1. sample collection and processing; 2. hybrid-capture enrichment sequencing; 3. bioinformatic analysis; 4. ARG-level data processing; 5. seasonality analysis.

Seasonal variation in influent samples was explored by hierarchical clustering of ARGs based on their relative seasonal variation profile. It used the R package *pheatmap v1.0.13* with “*Euclidean*” distance and linkage “*complete*”. Cluster selection, after visual inspection, was performed with the R *stats package v4.6.1 cutree* with parameter *k* = 2. For each ARG, a ratio (R) was calculated as the seasonal relative abundance divided by the annual median relative abundance of the same ARG. Values of R > 1 indicate seasonal enrichment relative to the annual median, whereas R < 1 indicates lower relative abundance. The median, rather than the mean, was used as the annual reference value because ARG abundance distributions were highly skewed, with a small number of dominant ARGs and a long tail of low-abundance genes (see Section 3.1); the median therefore provides a more robust estimate of the typical annual abundance level, less influenced by extreme high-abundance outliers. This normalisation approach was used to highlight seasonal fluctuations independently of absolute ARG abundance levels. Effluent samples were subsequently displayed using the same ARG order obtained from influent clustering in order to compare seasonal resistome patterns before and after wastewater treatment. The overall experimental and analytical workflow used for wastewater resistome characterisation is summarised in Figure 1.

## 3. Results

### 3.1. Resistome Overview: ARG Diversity and Abundance Structure

Across the eight wastewater samples collected over one year, a total of 743 ARGs were detected, corresponding to 26.6% of the 2786 targets included in the enrichment panel (Table S3). The number of detected ARGs per sample ranged from 408 to 625, with limited seasonal variation in total ARG richness. Most detected ARGs were shared between influent and effluent samples. Overall, 624 ARGs (84%) were detected in both wastewater stages, whereas 81 ARGs (11%) were detected exclusively in influents and 38 ARGs (5%) exclusively in effluents (Figure S2; Table S4). Relative-abundance analysis revealed a resistome structure characterised by a limited number of highly abundant ARGs coexisting with a large pool of low-abundance ARGs. Only 20 ARGs were classified as major ARGs (>1% relative abundance), yet together they accounted for approximately 60% of the median ARG-associated abundance across all eight wastewater samples (Table 1). In contrast, approximately 215 ARGs were classified as minor ARGs (0.1%–1% relative abundance), while more than 500 ARGs belonged to the ultra-minor category (<0.1% relative abundance) (Supplementary Table S5). Among major ARGs, only two exceeded 10% relative abundance: *ant(3″)*-variant genes (13.0%) and *bla*_oxa_-variant genes (11.6%). All remaining major ARGs ranged between 6.8% and 1.2% relative abundance (Table 1). Dominant ARGs were associated with multiple AACs, including aminoglycoside, beta-lactam, macrolide, tetracycline, and sulfonamide resistance. Overall, 35 AACs were represented across the dataset, including conventional antibiotic classes as well as broader categories such as multiclass and disinfectant-associated resistance.

### 3.2. Comparison of Influent and Effluent Resistomes Across Seasons

**Figure 2:**
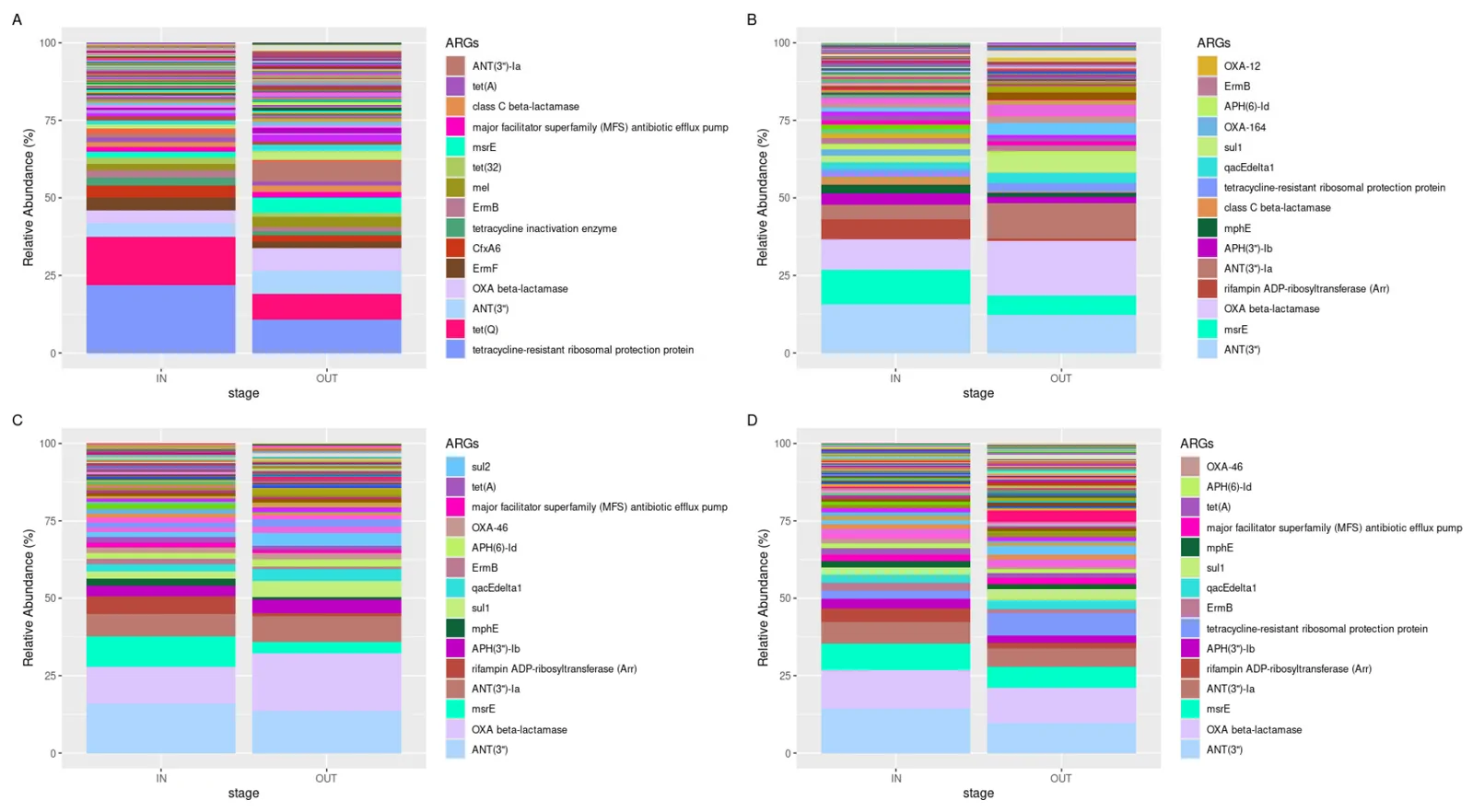
Relative abundance profile (rel. ab., %) of antibiotic resistance genes (ARGs) in influent (IN) and effluent (OUT) wastewater samples across the four seasons. Panels show winter (**A**), spring (**B**), summer (**C**) and fall (**D**). Within each panel, influent and effluent profiles are displayed side by side.

Across all seasons, influent and effluent samples exhibited broadly comparable resistome profiles, with the majority of ARGs detected in influents also identified in effluents (Figure 2). This overlap indicates substantial compositional similarity between influent and effluent resistomes. Despite this general similarity, differences in relative-abundance profiles were observed between influent and effluent samples. A subset of ARGs exhibited higher proportional abundance in effluents than influents. This shift mainly involved ARGs associated with aminoglycoside, macrolide and beta-lactam resistance, including recurrent dominant determinants such as *ant(3″)* variants, *msr*(E), *erm*(B) and *bla*_OXA_ variants.

Seasonal comparison revealed broadly similar resistome profiles across spring, summer and fall in both influent and effluent samples. In these seasons, the resistome was consistently shaped by ARGs associated with aminoglycoside, macrolide, streptogramin and beta-lactam resistance, including recurrent contributions from *ant(3″)* variants, *msr*(E), *aph(3″)-Ib* and *bla*_OXA_ variants. Additional stable contributions from genes such as *arr* (rifampin ADP-ribosyltransferase gene), *sul1*, and *tet* ribosomal protection protein, encoding tetracycline ribosomal protection proteins, were also observed.

In contrast, winter samples exhibited a distinct dominant profile characterised by relative contribution from *tet*(Q), *ant(3″)* variants, *bla*_OXA_ variants, *erm*(F), *cfxA6*, tetracycline-inactivating genes, *erm*(B), *mel, tet*(32) and *msr*(E), resulting in a resistome profile that differed from the recurrent pattern observed during the rest of the year.

These seasonal differences primarily reflected changes among highly abundant ARGs and did not fully capture the behaviour of the broader resistome, which was largely composed of low-abundance genes.

### 3.3. Distribution of ARGs Diversity and Abundance Across AACs-Level Analysis

**Figure 3:**
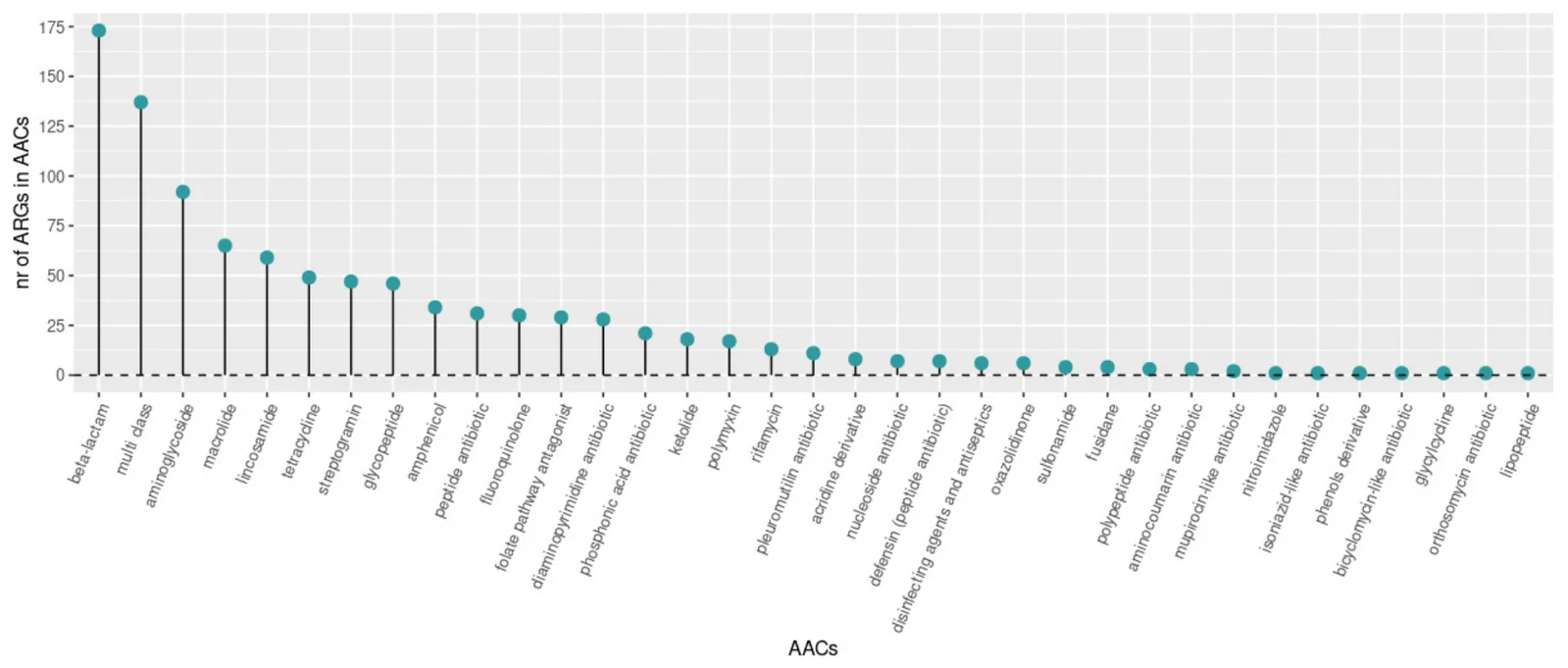
Distribution of antibiotic resistance genes (ARGs) across AACs over one year of wastewater monitoring. The x-axis shows the different AACs, while the y-axis reports the number of ARGs detected within each category.

Analysis at the Antimicrobial Agent Categories (AACs) level revealed broad functional diversity within the wastewater resistome. Across the eight samples, a total of 35 AACs were represented (Figure 3), including both conventional antibiotic classes and broader Antimicrobial Agent Categories. The distribution of ARGs across AACs was highly uneven. Categories associated with beta-lactam, multi-class, aminoglycoside, macrolide, lincosamide, tetracycline, streptogramin and glycopeptide resistance contained the largest number of detected ARGs, whereas several other AACs were represented by only a limited number of ARGs. Among all categories, beta-lactam-associated resistance showed the highest diversity, with approximately 180 detected ARGs. Overall, only a limited number of AACs accounted for most of the detected ARG diversity (Figure 3).

**Figure 4:**
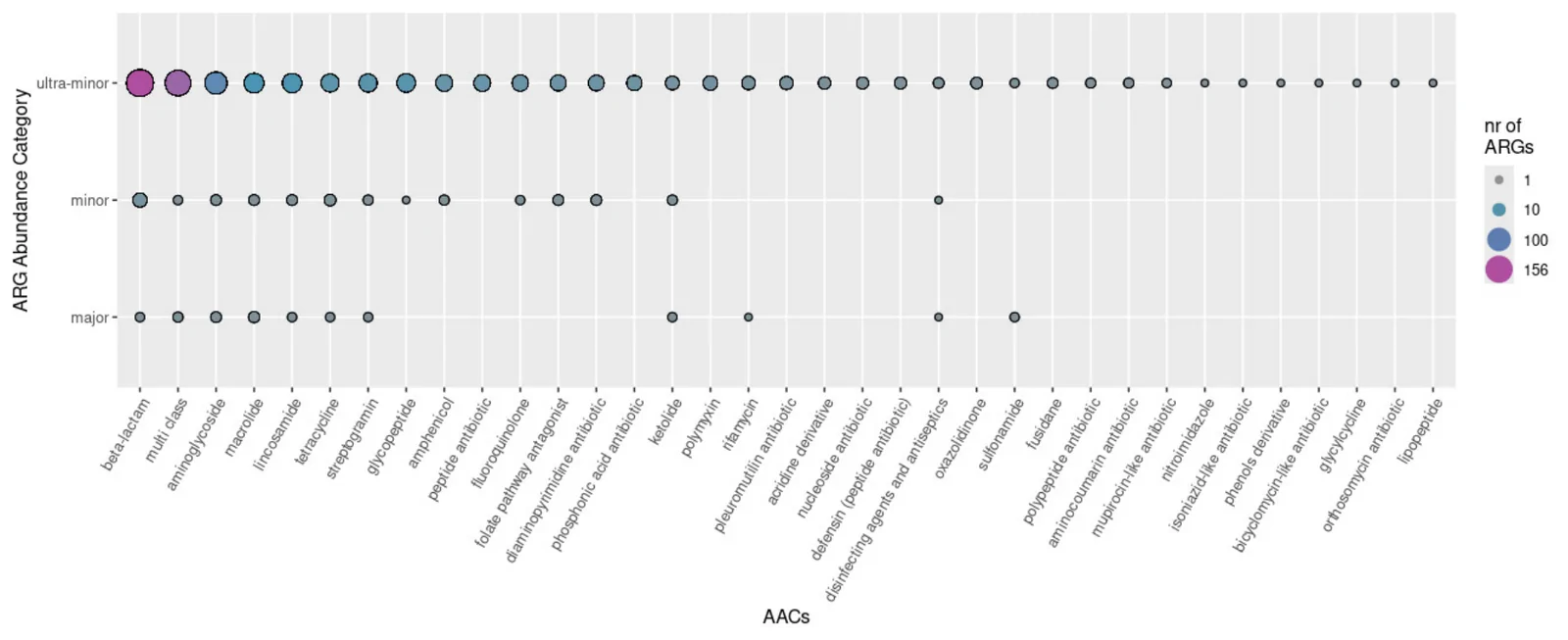
Distribution of ARGs across Antimicrobial Agent Categories (AACs) according to relative-abundance categories. ARGs were classified as major (>1%), minor (0.1–1%) or ultra-minor (<0.1%). The x-axis reports AACs, and the y-axis shows the three relative-abundance categories.

Based on relative-abundance analysis (major, minor, ultra-minor), the contribution of individual ARGs within each AAC was evaluated (Figure 4). Only 11 AACs included major ARGs that, in most cases, were fewer than ten. Fourteen AACs included minor ARGs, whereas all 35 AACs contained ultra-minor ARGs, often in substantial numbers. Notably, AACs characterised by high ARG diversity were not necessarily those contributing most to the overall relative abundance. Instead, the resistome was dominated by a limited number of highly abundant ARGs distributed across multiple AACs. Overall, the AAC-level distribution confirmed that the majority of ARG diversity was represented by low-abundance ARGs spanning nearly all AACs, whereas only a limited number of ARGs contributed substantially in terms of relative abundance to the total resistome composition.

### 3.4. Seasonal Variation and Cluster Analysis of the Wastewater Resistome

**Figure 5:**
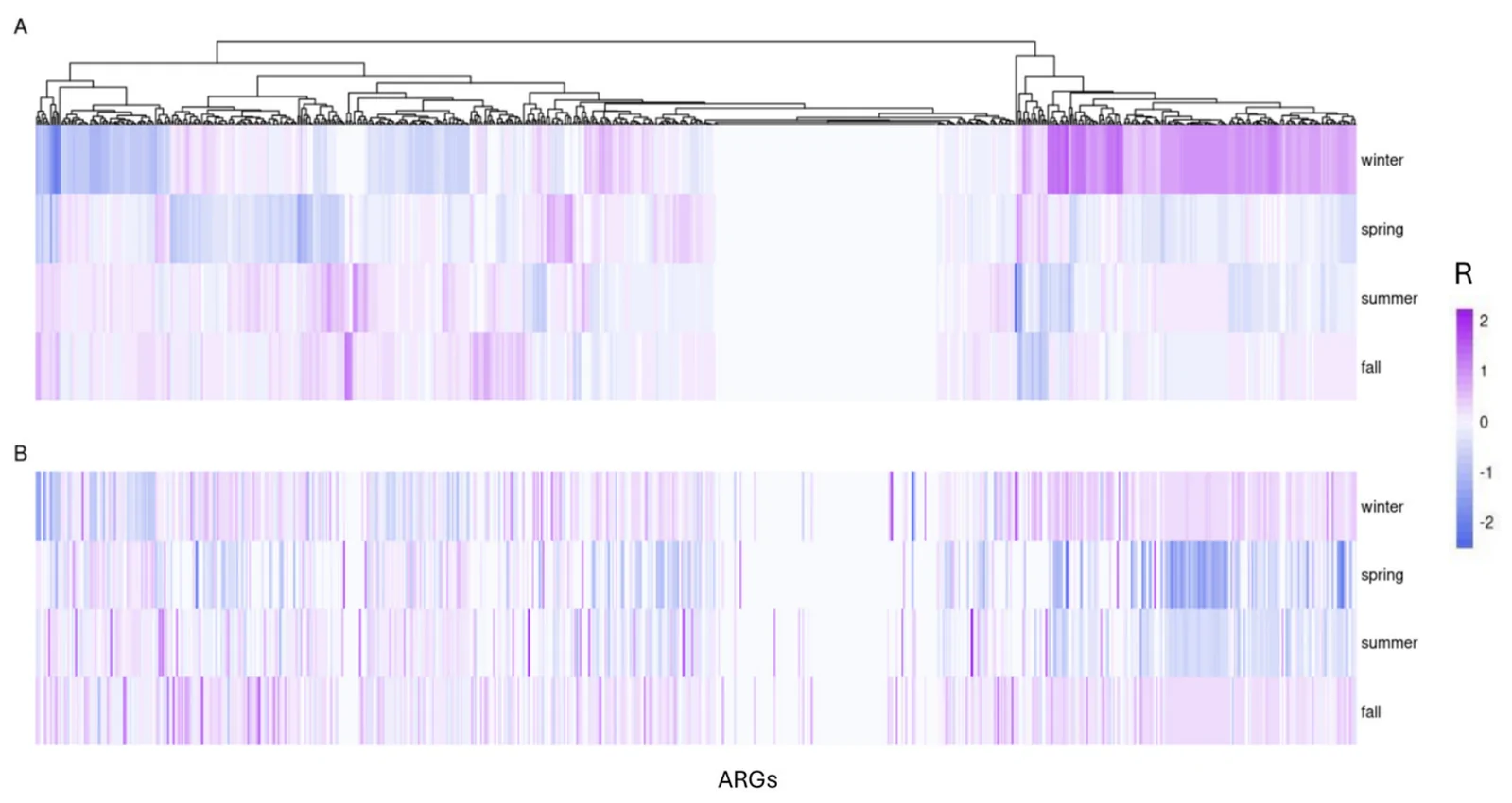
Seasonal clustering of antibiotic resistance genes (ARGs) in influent and effluent wastewater samples. (**A**) Heatmap showing seasonal variation in ARG abundance in influent samples, expressed as the ratio between seasonal abundance and annual median abundance, with hierarchical clustering applied to genes. (**B**) Heatmap of effluent samples displayed using the same gene order obtained from influent clustering, allowing direct comparison of seasonal patterns before and after treatment.

As shown previously in the seasonal relative-abundance profiles (Figure 2A–D), the overall structure of the wastewater resistome remained broadly stable throughout the year. However, the identity and relative contribution of the ARGs varied across seasons. To investigate seasonal dynamics beyond dominant ARGs, hierarchical clustering was therefore applied using each ARG’s seasonal abundance relative to its annual median abundance (R). In influent samples (Figure 5A), most ARGs displayed limited seasonal variation and grouped into broader, stable clusters. Nevertheless, a distinct subset of ARGs showed a clear, coordinated winter-associated enrichment, forming a second, smaller cluster. This winter-associated trend signal involved predominantly low-abundance ARGs. When the same gene order, derived by clustering, was applied to effluent samples (Figure 5B), the coordinated winter-associated pattern became markedly less evident. Although the same ARGs remained detectable, their seasonal trajectories appeared more heterogeneous. Overall, 191 ARGs (26% of all detected genes) showed winter-associated enrichment, spanning 27 of the 35 AACs (77%) (Supplementary Figure S3). Only a limited fraction of these ARGs reached high relative abundance during winter and were mainly associated with tetracycline resistance, explaining the distinctive seasonal shift observed among dominant ARGs. Most winter-enriched ARGs instead belonged to the minor and ultra-minor abundance categories and were distributed across multiple AACs. Taken together, these results reveal a structured winter-associated enrichment affecting a substantial portion of the influent resistome, which is not retained after wastewater treatment.

## 4. Discussion

Understanding the structure and dynamics of ARGs in wastewater is becoming increasingly important within the broader One Health framework, particularly as wastewater-based surveillance is progressively integrated into environmental AMR monitoring strategies. However, most current environmental surveillance approaches rely on targeted qPCR or dPCR assays focused on a relatively limited number of clinically prioritised ARGs. While highly sensitive and suitable for routine quantification, these methods provide only a partial representation of wastewater resistomes and may overlook the extensive diversity of low-abundance resistance determinants circulating within complex environmental microbial communities. In this context, the present study demonstrates that hybrid-capture targeted enrichment sequencing can provide a substantially broader and more resolved characterisation of wastewater resistomes across Antimicrobial Agent Categories (AACs), abundance levels and seasonal conditions. To date, the application of hybrid-capture enrichment panels for wastewater resistome surveillance remains extremely limited, with only a few studies having explored this approach in environmental wastewater matrices [12,13]. Consequently, the present study contributes to a still largely unexplored field and provides one of the first evaluations of this technology for wastewater resistome characterisation across treatment stages and seasons. By targeting 2786 resistance ARGs, the enrichment strategy enabled the detection of 743 ARGs across influent and effluent wastewater samples collected over one year. Beyond identifying dominant resistance ARGs, the approach revealed a large pool of low-abundance ARGs distributed across multiple AACs, providing a more comprehensive view of resistome organisation than would be achievable using conventional targeted surveillance strategies alone. One of the main findings of this study was the strongly uneven organisation of the wastewater resistome. Relative-abundance analyses consistently revealed a structure characterised by a limited number of highly abundant ARGs coexisting with a much broader diversity of low-abundance ARGs. Only a small subset of ARGs accounted for most of the sequencing reads output, whereas hundreds of ARGs were detected at *ultra-minor* abundance levels across nearly all AACs. In this context, dominant ARGs likely reflect persistent resistance determinants continuously introduced into the sewer system through the contributing population, whereas low-abundance ARGs may originate from heterogeneous microbial populations, transient colonisers, rare taxa or mobile genetic elements circulating at lower frequencies within the wastewater environment, or resistance determinants associated with antimicrobials whose use may exert a comparatively lower selective pressure in this setting. Importantly, this uneven organisation was also reflected at the functional level. Although 35 AACs were represented across the dataset, ARG diversity was highly unevenly distributed among them. AACs, including beta-lactam, multiclass, aminoglycoside, macrolide, tetracycline, and glycopeptide, contained the highest number of detected ARGs, whereas several other AACs were represented by only a limited number of ARGs. At the same time, the most abundant ARGs were distributed across multiple AACs rather than concentrated within a single dominant one, indicating that the dominant fraction of the wastewater resistome is sustained by a heterogeneous combination of resistance mechanisms. In this context, the high number of glycopeptide ARGs requires careful interpretation. In wastewater samples, the enrichment approach is not detecting multiple independent vancomycin resistance ARGs as separate entities; rather, it captures different components of the same glycopeptide-resistance operons. Because glycopeptide resistance is mediated by highly coordinated genetic clusters, the panel recovers not only the core resistance determinants, but also associated synthesis (*vanH*), cleavage (*vanX*), and regulatory (*vanR*/*vanS*) genes. Consequently, the elevated ARG count does not necessarily indicate a wide diversity of independent resistance mechanisms; rather, it reflects the detection of complete or partially complete functional resistance operons circulating within the wastewater microbial community. A major advantage of the enrichment-based approach emerged from its ability to characterise a much broader resistome dynamics that would likely remain unresolved using conventional PCR-based surveillance focused on a limited number of targets. The use of clustering analysis revealed a coordinated winter-associated enrichment involving 191 ARGs distributed across 27 AACs. Notably, most of these winter-enriched ARGs belonged to the *minor* and *ultra-minor* abundance groups, demonstrating that relevant ecological dynamics may remain hidden when analyses focus exclusively on dominant ARGs or clinically prioritised resistance markers. The winter-associated enrichment observed in influent wastewater likely reflects the combined effect of multiple seasonal drivers, including changes in antibiotic consumption, infection incidence, human microbiota composition and wastewater microbial ecology during colder months. In particular, the increased contribution of tetracycline-associated ARGs during winter suggests that seasonal antibiotic usage patterns may influence not only dominant ARGs but also broader, low-abundance resistome components. This sharp winter peak aligns with global epidemiological and pharmacological data, which report tetracyclines as heavily used first-line broad-spectrum antibiotics for human seasonal infections, with a daily human consumption of 23 kg globally, and veterinary therapy, exceeding 2500 tons annually in Europe. Crucially, because approximately 75% of the administered tetracycline is excreted in its parent, active form, massive seasonal amounts enter the sewage system [15]. According to the Italian Medicines Agency (AIFA) National Report on Antibiotic Use 2023 [16], Central Italy is characterised by substantial use of broad-spectrum antibiotics and pronounced seasonal fluctuations in consumption, with higher prescription rates during the autumn and winter months coinciding with increased incidence of respiratory infections. Penicillins remain the most prescribed antibiotic class, followed by cephalosporins and macrolides in human medicine, while tetracyclines continue to represent a major component of veterinary antimicrobial use. These consumption patterns may contribute to the continuous introduction of antibiotic residues and resistant bacteria into wastewater systems and could partially explain the winter-associated enrichment of tetracycline-related ARGs observed in this study, supporting the hypothesis that seasonal antibiotic use contributes to shaping wastewater resistome composition. This high prescription volume of beta-lactam antibiotics in human medicine is also consistent with the substantial and stable abundance of *bla*OXA-variant genes observed across the wastewater resistome throughout the year (Table 1), suggesting that sustained clinical beta-lactam use may similarly contribute to the persistent dominance of beta-lactam resistance determinants in this setting. Based on the AIFA National Report 2023 [16], Italy continues to rely heavily on broad-spectrum β-lactams; the ratio of broad-spectrum to narrow-spectrum antibiotic use was 13.6 in 2023, compared with a European average of 5.5. The extensive β-lactam use contributes to increased selective pressure and the emergence of β-lactam resistance. Specifically, for β-lactam resistance, Regions with higher overall antibiotic consumption consistently show higher resistance rates among important bacterial pathogens. The relationship is particularly evident for antibiotics that exert strong selective pressure, including penicillins, amoxicillin/clavulanate, and third-generation cephalosporins, all belonging to the β-lactam class. However, in the absence of catchment-specific, temporally resolved antibiotic consumption data, both associations remain qualitative and should not be interpreted as evidence of a direct quantitative or causal relationship.

Similarly, data from the national antibiotic-resistance surveillance system AR-ISS [17] reveal a regional AMR profile largely consistent with national trends, with sustained circulation of third-generation cephalosporin- and carbapenem-resistant Enterobacterales, along with methicillin-resistant *Staphylococcus aureus* (MRSA) and vancomycin-resistant *Enterococcus faecium* (VR*E-faecium*) in healthcare settings. The predominance of beta-lactam-, glycopeptide-, macrolide- and multidrug-resistance determinants observed in the wastewater resistome is consistent with the resistance patterns documented by national AMR surveillance on invasive infections. Together, these findings suggest that the wastewater resistome can act as valuable environmental proxies for AMR circulation within the population, providing complementary information to AMR clinical surveillance systems and strengthening integrated One Health monitoring strategies. Consequently, integrating wastewater-based resistome monitoring with antibiotic consumption data and AMR surveillance indicators could provide a more comprehensive assessment of AMR circulation within a One Health framework. Another relevant observation was the marked difference between influent and effluent seasonal organisation. Although most ARGs detected in influents remained detectable after treatment, the coordinated winter-associated clustering pattern observed upstream became substantially less structured in effluent samples. This suggests that wastewater treatment may alter coordinated ecological relationships among ARGs even when individual ARGs persist within the treated effluent resistome. Importantly, these findings should be interpreted cautiously, as the present study relied on relative abundance rather than absolute quantification and therefore cannot directly assess ARG removal efficiency. From a surveillance perspective, these findings support the growing interest in wastewater-based AMR monitoring as a complementary component of integrated One Health surveillance frameworks. Recent international initiatives, including the revised European Urban Wastewater Treatment Directive, increasingly recognise wastewater as a valuable source of population-level information on antimicrobial resistance circulation. However, it is important to critically evaluate the direct applicability of targeted enrichment sequencing within the strict context of routine regulatory monitoring. While this approach offers an unprecedented, enhanced and broader view of the wastewater resistome, its cost, turnaround time, and technical complexity might limit its feasibility as a standard primary screening tool for compliance with the Urban Wastewater Treatment Directive on a large scale. Nevertheless, although it may not represent the primary tool for standard regulatory monitoring, enrichment-based sequencing approaches may offer important advantages for environmental surveillance by simultaneously capturing dominant clinically relevant ARGs together with low-abundance resistance determinants that may represent emerging or previously overlooked resistance reservoirs. A key strength of hybrid-capture enrichment approaches lies precisely in their exceptional adaptability and customizability. Although the present study employed a commercially available predefined panel, probe sets can be customised to include a broader spectrum of ARGs, or emerging targets of specific epidemiological or regulatory interest. This flexibility may represent an important advantage for future environmental AMR surveillance frameworks, where monitoring priorities are expected to evolve over time in response to emerging resistance threats, regional epidemiological drivers and regulatory requirements. Moreover, enrichment strategies may be particularly advantageous when analysing low-biomass environmental matrices or investigating rare resistance determinants that would otherwise remain below detection thresholds using conventional untargeted approaches.

Several limitations of this study should be acknowledged. First, the investigation was conducted on a single WWTP and included a relatively limited number of seasonal samples, restricting the generalisability of the findings and limiting the possibility of assessing inter-annual variability. Second, the study relied on targeted enrichment rather than untargeted metagenomics and therefore could not capture novel or unexpected ARGs outside the panel design; any resistance determinant not represented among the 2786 targeted genes remains invisible to this approach regardless of its actual abundance. This also affects the interpretation of the overall detection rate: only 743 of the 2786 targeted ARGs (26.6%) were detected, and non-detection of the remaining targets should not be interpreted as evidence of genuine absence, as it may instead reflect abundance below the assay’s detection threshold, insufficient sequencing depth, or variability in probe-capture efficiency across gene families. Distinguishing these possibilities would require complementary approaches, such as targeted qPCR/dPCR confirmation or parallel shotgun metagenomic sequencing, which were beyond the scope of this proof-of-concept study. Third, the absence of parallel metadata, including antibiotic consumption data, physicochemical parameters, microbial community composition, or clinical AMR surveillance data, limited the possibility of identifying the specific drivers underlying the observed seasonal patterns. Relatedly, the WWTP catchment is known to receive discharges from at least three major hospitals alongside domestic wastewater, but detailed information on the relative contribution of these and other potential sources (e.g., industrial or agricultural discharges) was not available; because sampling was based on composite influent collected at the plant inlet, without source-specific sampling or molecular source-tracking, this study cannot disentangle the contribution of individual inflow sources to the observed resistome composition, nor establish a causal link between specific sources and ARG dissemination. Finally, because the analysis relied on relative-abundance metrics derived from enrichment sequencing data, direct quantitative comparisons of ARG loads and treatment removal dynamics should be interpreted with caution.

Despite these limitations, this proof-of-concept study demonstrates that hybrid-capture targeted enrichment sequencing can provide a highly resolved and ecologically informative characterisation of wastewater resistomes across treatment stages and seasons. Beyond identifying dominant ARGs, the approach revealed extensive low-abundance ARG diversity and uncovered coordinated seasonal dynamics that would likely remain unresolved using conventional targeted surveillance approaches.

## 5. Conclusions

This proof-of-concept study provides one of the first longitudinal evaluations of hybrid-capture targeted enrichment sequencing for wastewater resistome surveillance across treatment stages and seasons. Applying a panel targeting 2786 ARGs to influent and effluent samples collected over one year, we detected 743 ARGs, 84% of which were shared between treatment stages, revealing a strongly uneven resistome structure in which a limited number of highly abundant ARGs coexisted with a much broader pool of low-abundance genes distributed across 35 Antimicrobial Agent Categories. Seasonal analysis further uncovered a structured winter-associated enrichment, particularly involving tetracycline-related ARGs, that was evident in influents but markedly less defined in effluents. Overall, these findings demonstrate that targeted enrichment sequencing can capture both dominant and low-abundance ARGs beyond the reach of conventional PCR-based monitoring, supporting the potential value of enrichment-based sequencing strategies for future environmental AMR surveillance and highlighting the importance of considering the broader low-abundance resistome when investigating the environmental dynamics of antimicrobial resistance.

## Figures and Tables

**Table 1:** Major ARGs (>1% relative abundance) identified across the complete eight-sample wastewater resistome dataset. Median TPM and relative abundance are calculated across the complete eight-sample wastewater samples.

ARGs	AACs	MedianTPM	Rel. Abundance (%)
*ant(3″)* variants	aminoglycoside	130,372.5	13.0
*bla*_OXA_ variants	beta-lactam	116,172.2	11.6
*ant(3* *″* *)-Ia*	aminoglycoside	68,181.6	6.8
*msr*(E)	macrolide; streptogramin	64,671.3	6.5
*aph(3* *″* *)-Ib*	aminoglycoside	28,068.0	2.8
*tet* ribosomal protection protein	tetracycline	27,295.6	2.7
*sul1*	sulfonamide	26,657.2	2.7
*qacEdelta1*	disinfecting agents and antiseptics	23,840.9	2.4
*erm*(B)	macrolide; lincosamide; ketolide; streptogramin	17,941.1	1.8
Major Facilitator Superfamily-type efflux pump	multiclass	16,770.3	1.7
Class C beta-lactamase	beta-lactam	15,312.5	1.5
*sul2*	sulfonamide	15,205.6	1.5
*arr*	rifamycin	14,890.5	1.5
*aph(6)-Id*	aminoglycoside	14,771.5	1.5
*aadA7*	lincosamide; aminoglycoside	14,770.5	1.5
*tet*(A)	tetracycline	14,374.3	1.4
*bla*_OXA-46_	beta-lactam	14,133.8	1.4
*mph*(E)	macrolide	13,770.2	1.4
*mph*(A)	macrolide; ketolide	12,423.0	1.2
Macrolide esterase	macrolide	11,524.9	1.2

## Data Availability

The original contributions presented in this study are included in the article/Supplementary Materials. Further inquiries can be directed to the corresponding author.

## Supplementary Materials

The following supporting information can be downloaded at: https://www.mdpi.com/article/10.3390/toxics14080650/s1, Table S1. List of ARGs present in the QIAseq xHYB AMR Panel (see Excel file); Table S2. Categories of Antimicrobial Agents; Figure S1. Overview of the Experimental Workflow; Table S3. Resistance abundance table obtained with FRSB (see Excel file); Figure S2. Venn diagram of ARGs found in wastewater. A. Number of ARGs (81) detected only in influent wastewater samples; B. Number of ARGs (38) detected only in effluent wastewater samples. AB. Number of ARGs (624) shared between influent and effluent wastewater samples; Table S4. List of ARGs detected in this study (see Excel file); Figure S3. Number of ARGs belonging to each Antimicrobial Agent Category within the winter-enriched cluster compared to the full annual dataset. The blue points show the total number of ARGs detected in each AAC over the full year, while the purple points indicate how many of those ARGs belong specifically to the winter-enriched cluster.

## Abbreviations

The following abbreviations are used in this manuscript:

WWTP	Wastewater Treatment Plant
ARG	Antimicrobial Resistance Gene
AAC	Antimicrobial Agent Category

## References

[1] Karkman A., Do T.T., Walsh F., Virta M.P.J. (2018). Antibiotic-Resistance Genes in Waste Water. Trends Microbiol..

[2] Nguyen A.Q., Vu H.P., Nguyen L.N., Wang Q., Djordjevic S.P., Donner E., Yin H., Nghiem L.D. (2021). Monitoring antibiotic resistance genes in wastewater treatment: Current strategies and future challenges. Sci. Total Environ..

[3] Pazda M., Kumirska J., Stepnowski P., Mulkiewicz E. (2019). Antibiotic resistance genes identified in wastewater treatment plant systems—A review. Sci. Total Environ..

[4] World Health Organization (2021). WHO strategic priorities on antimicrobial resistance: Preserving antimicrobials for today and tomorrow. https://iris.who.int/handle/10665/351719.

[5] European Commission (2017). A European One Health Action Plan against Antimicrobial Resistance (AMR). https://health.ec.europa.eu/publications/european-one-health-action-plan-against-antimicrobial-resistance-amr_en.

[6] European Commission (2022). Directive of the European Parliament and of the Council concerning urban wastewater treatment (recast). https://eur-lex.europa.eu/legal-content/EN/TXT/?uri=CELEX%3A52022PC0541.

[7] Bonanno Ferraro G., Bonomo C., Brandtner D., Mancini P., Veneri C., Briancesco R., Coccia A.M., Lucentini L., Suffredini E., Bongiorno D. (2024). Characterisation of microbial communities and quantification of antibiotic resistance genes in Italian wastewater treatment plants using 16S rRNA sequencing and digital PCR. Sci. Total Environ..

[8] Hultman J., Tamminen M., Pärnänen K., Cairns J., Karkman A., Virta M. (2018). Host range of antibiotic resistance genes in wastewater treatment plant influent and effluent. FEMS Microbiol. Ecol..

[9] Li J., Cheng W., Xu L., Strong P.J., Chen H. (2015). Antibiotic-resistant genes and antibiotic-resistant bacteria in the effluent of urban residential areas, hospitals, and a municipal wastewater treatment plant system. Environ. Sci. Pollut. Res..

[10] Rafraf I.D., Lekunberri I., Sànchez-Melsió A., Aouni M., Borrego C.M., Balcázar J.L. (2016). Abundance of antibiotic resistance genes in five municipal wastewater treatment plants in the Monastir Governorate, Tunisia. Environ. Pollut..

[11] Pant A., Shahadat M., Ali S.W., Ahammad S.Z. (2022). Removal of antimicrobial resistance from secondary treated wastewater—A review. J. Hazard. Mater. Adv..

[12] Baba H., Kuroda M., Sekizuka T., Kanamori H. (2023). Highly sensitive detection of antimicrobial resistance genes in hospital wastewater using the multiplex hybrid capture target enrichment. mSphere.

[13] Sekizuka T., Yamaguchi N., Kanamori H., Kuroda M. (2023). Multiplex hybrid capture improves the deep detection of antimicrobial resistance genes from wastewater treatment plant effluents to assess environmental issues. Microb. Drug Resist..

[14] Alcock B.P., Huynh W., Chalil R., Smith K.W., Raphenya A.R., Wlodarski M.A., Edalatmand A., Petkau A., Syed S.A., Tsang K.K. (2023). CARD 2023: Expanded curation, support for machine learning, and resistome prediction at the Comprehensive Antibiotic Resistance Database. Nucleic Acids Res..

[15] Amangelsin Y., Semenova Y., Dadar M., Aljofan M., Bjørklund G. (2023). The impact of tetracycline pollution on the aquatic environment and removal strategies. Antibiotics.

[16] The Medicines Utilisation Monitoring Centre (2025). National Report on Antibiotics Use in Italy.

[17] Iacchini S., Boros S., Pezzotti P., Errico G., Del Grosso M., Camilli R., Giufrè M., Pantosti A., Maraglino F., Palamara A.T. (2024). AR-ISS: Sorveglianza Nazionale Dell’AntibioticoResistenza; Dati 2023 (Rapporti ISS Sorveglianza RIS-5/2024).

