# Supplementary material for: Targeted Enrichment Sequencing for Wastewater Resistome Surveillance: A Proof-of-Concept Study Across Treatment Stages and Seasons
Source: Toxics. 2026 Jul 24;14(8):650. doi: 10.3390/toxics14080650 (PMC13517800; doi:10.3390/toxics14080650)
Supplement: Supplementary file 1 [file toxics-14-00650-s001.zip › toxics-4409720-Supplementary Material.pdf]

Supplementary files

Table S1. List of ARGs present in the QIAseq xHYB AMR Panel (see Excel file).

Table S2. Categories of Antimicrobial Agents.

Categories of Antimicrobial Agents
Acridine dye
Aminoglycoside antibiotic
Ampicillin
Bacitracin
Benzylpenicillin
Bicyclomycin
Carbapenem
Cephalosporin
Cephem
Ciprofloxacin
Defensin
Disinfecting agents and intercalating dyes
Factumycin
Fosfomycin
Gentamicin C
Glycylcycline
Kasugamycin
Lincosamide antibiotic
Meropenem
Metronidazole
Mupirocin
Novobiocin
Oleandomycin

Penicillin
Phenicol antibiotic
Pleuromutilin
Spectinomycin
Streptogramin A antibiotic
Acriflavine
Amoxicillin
Apramycin
BAL-30072
Beta-lactam antibiotic
Capreomycin
Ceftazidime
Cephamycin
Chloramphenicol
Clindamycin
Dihydropyrimidine antibiotic
Erythromycin
Fluoroquinolone antibiotic
Fusidic acid
Glycopeptide antibiotic
Kanamycin A
Lincomycin
Macrolide antibiotic
Methicillin
Microcin J25
Neomycin
Nucleoside antibiotic
Penam
Peptide antibiotic

Plazomicin
Rifampicin antibiotic
Spiramicin
Streptogramin B antibiotic

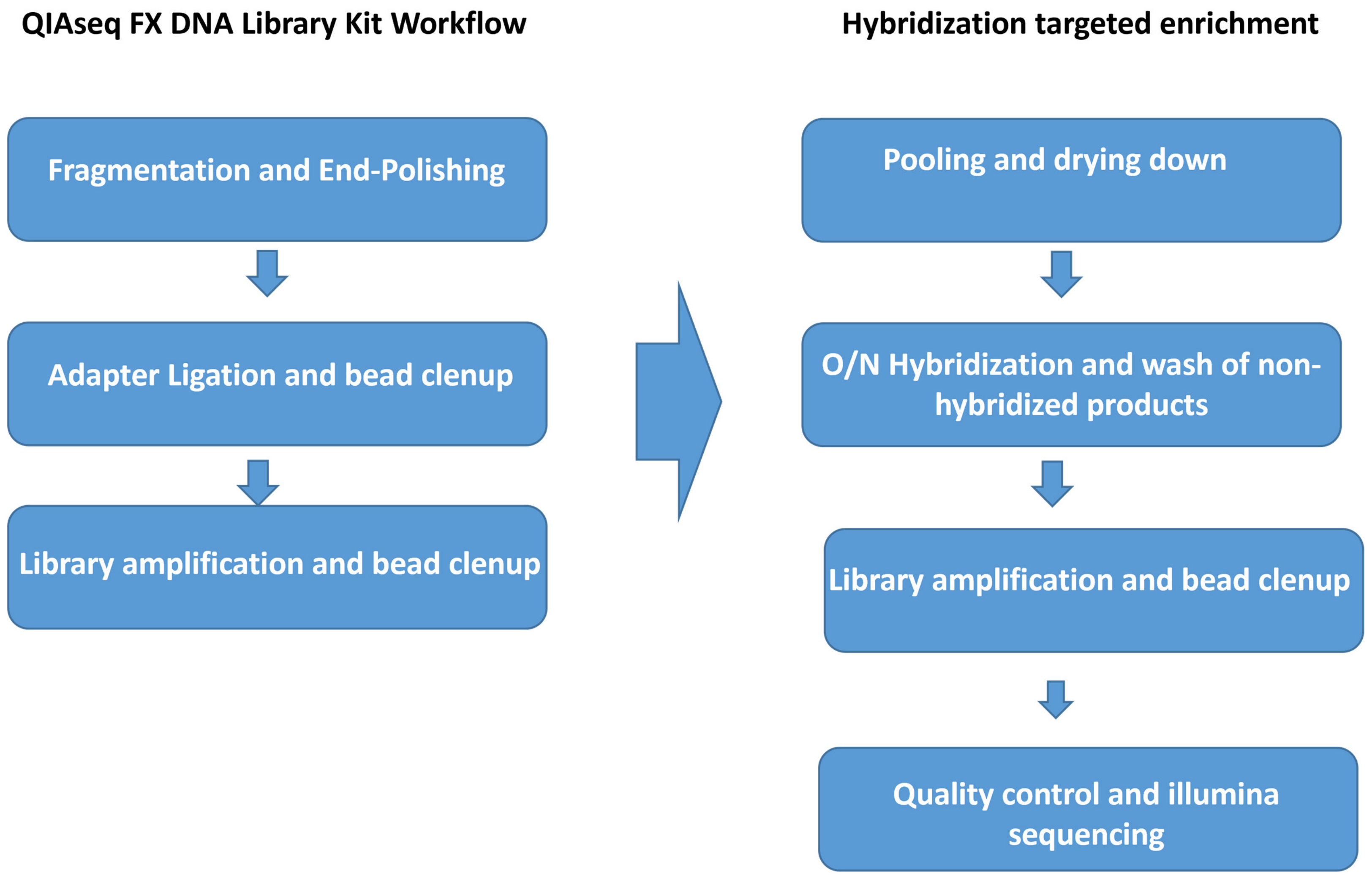

Figure S1. Overview of the Experimental Workflow.

Table S3. Resistance abundance table obtained with FRSB (see Excel file).

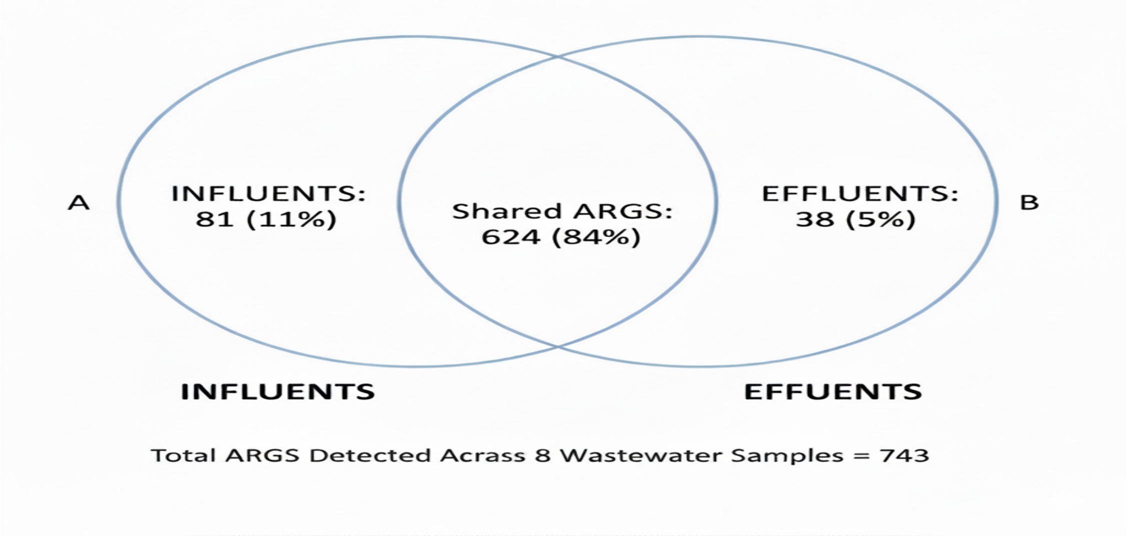

Figure S2. Venn diagram of ARGs found in wastewater. A. Number of ARGs (81) detected only in influents wastewater samples; B. Number of ARGs (38) detected only in effluents wastewater samples. AB. Number of ARGs (624) shared between influents and effluents wastewater samples.

Table S4. List of ARGs detected in this study (see Excel file).

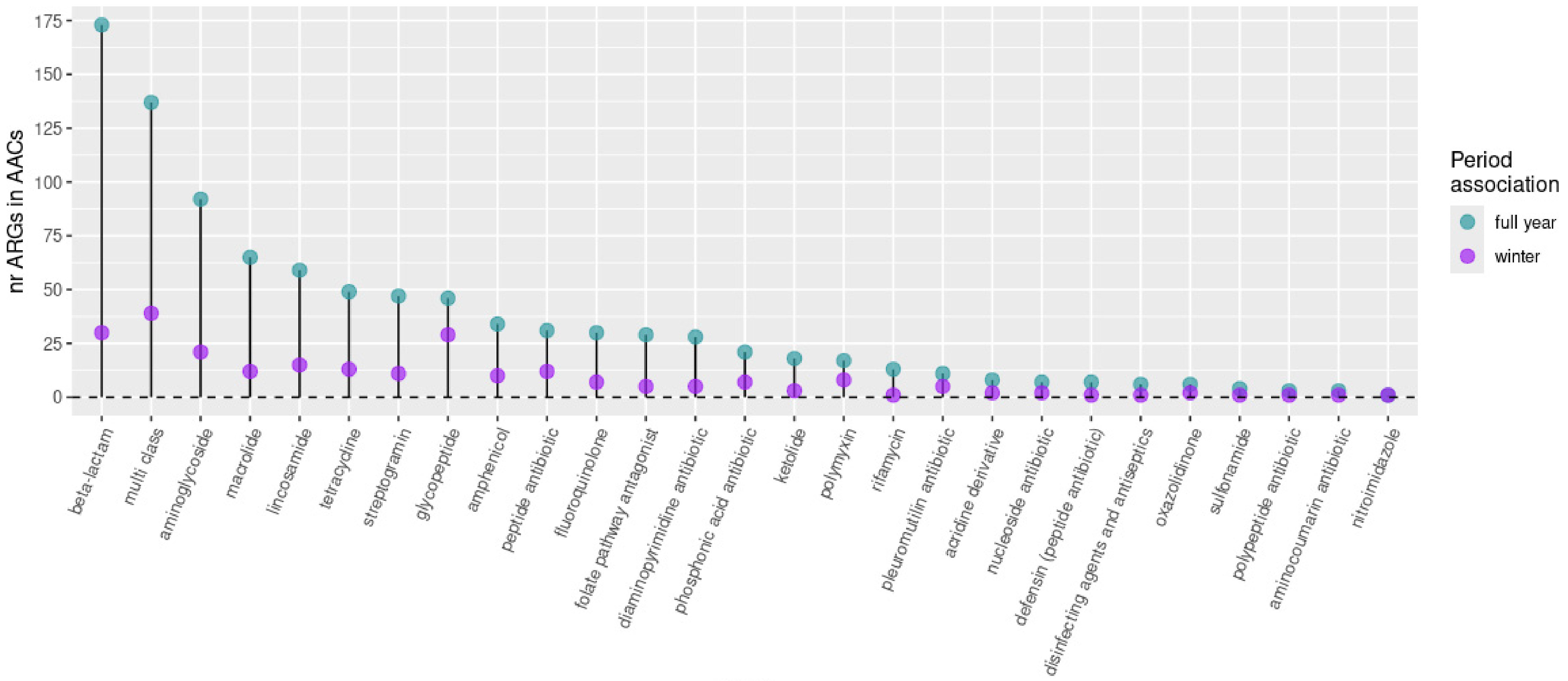

Figure S3. Number of ARGs belonging to each Antimicrobial Agent Categories within the winter-enriched cluster compared to the full annual dataset. The blue points show the total number of ARGs detected in each AAC over the full year, while the purple points indicate how many of those ARGs belong specifically to the winter-enriched cluster.
